# A comparative analysis of deep learning models for assisting in the diagnosis of periapical lesions in periapical radiographs

**DOI:** 10.1186/s12903-025-06104-0

**Published:** 2025-05-26

**Authors:** Jian Liu, Chaoran Jin, Xiaolan Wang, Kexu Pan, Zhuoyang Li, Xinxuan Yi, Yu Shao, Xiaodong Sun, Xijiao Yu

**Affiliations:** 1 School of Stomatology, Shandong Second Medical University, Weifang, 261053 Shandong China; 2 Department of Endodontics, Central Laboratory, Jinan Stomatological Hospital, Jinan Key Laboratory of oral tissue regeneration, Shandong Provincial Key Medical and Health Laboratory of Oral Diseases and Tissue Regeneration, Jinan, 250001 Shandong Province China; 3 Shandong Xintai Huizhi Health and Medical Big Data Co., Ltd., Jinan, 250001 Shandong Province China; 4 Shungeng Branch, Central Laboratory, Jinan Stomatological Hospital, Jinan Key Laboratory of oral tissue regeneration, Shandong Provincial Health Commission Key Laboratory of Oral Diseases and Tissue Regeneration, Jinan, 250001 Shandong Province China

**Keywords:** ConvNeXt, ResNet34, Deep learning, Periapical radiographs, Periapical lesion

## Abstract

**Purpose:**

Numerous studies have investigated the use of convolutional neural network (CNN) models for detecting periapical lesions(PLs). However, limited research has focused on evaluating their potential in assisting clinicians with diagnosis. This study aims to utilize two deep learning(DL) models, ConvNeXt and ResNet34, to aid novice dentists in the detection of PLs on periapical radiographs (PRs). By assessing the diagnostic support provided by these models, this research seeks to promote the clinical application of DL in dentistry.

**Materials and methods:**

In this study, 1,305 PRs were gathered and then split into a training set of 1,044 images and a validation set of 261 images, following an 80/20 ratio. The model’s effectiveness was assessed using various measures, including precision, sensitivity, F1 score, specificity, accuracy, and the area under the curve (AUC). To evaluate the impact of the model on diagnostic performance by novice dentists, we used an additional set of 800 individual teeth PRs, which were not included in the training or validation sets. The diagnostic performance and time of three novice dentists were measured both with and without model assistance.

**Results:**

The precision of ConvNeXt was 85.93%, with an F1 score of 0.92, accuracy of 91.25%, sensitivity of 98.49%, specificity of 84.11%, and an AUC of 0.9693, outperforming ResNet34 across all metrics. In comparison, ResNet34 achieved a precision of 83.08%, an F1 score of 0.84, accuracy of 81.63%, sensitivity of 84.38%, specificity of 78.13%, and an AUC of 0.8988. In the model-assisted diagnosis phase, both ConvNeXt and ResNet34 improved the diagnostic performance of novice dentists. With the help of ConvNeXt, the average AUC of three dentists increased from 0.88 to 0.94, while with ResNet34, the average AUC of the three dentists improved from 0.88 to 0.91. ConvNeXt performed better than ResNet34 (*p* < 0.05). Additionally, ConvNeXt reduced the average diagnostic time of the three dentists from 178.8 min to 141.9 min, while ResNet34 reduced the average diagnostic time from 178.8 min to 153.6 min.

**Conclusion:**

ConvNeXt significantly improved the diagnostic performance of novice dentists and reduced the time required for diagnosis, thereby enhancing clinical efficiency in both diagnosis and treatment. This model shows potential for application in dental clinics or educational institutions where experienced specialists are limited, but there is a large presence of novice, less-experienced dentists.

## Introduction

Apical periodontitis is a prevalent inflammatory condition in dentistry, with a prevalence of 23-31% in the population [1], posing a substantial global burden [2]. In radiology, periapical periodontitis manifests as an apical radiolucent lesion caused by the widening of the periodontal ligament space [3], also known as an apical lesion. Apical lesions are generally asymptomatic in their early stages and are typically discovered during radiographic follow-ups of teeth that have undergone root canal treatment [4]. This frequently leads to missed opportunities for optimal treatment, leading to the inability to preserve the tooth, and in some cases, causing severe complications [5]. Cone Beam Computed Tomography (CBCT), panoramic radiographs, and PRs are all common clinical methods for detecting PLs. The high cost and radiation dose of CBCT restrict its clinical application [6]. The PR is the overall standard for detecting changes in radiolucent periapical alterations, especially for diagnosing periapical periodontitis [7]. It presents a cost-effective and efficient diagnostic approach, delivering detailed visualization of the affected tooth along with the surrounding tissues [8]. In comparison to panoramic radiographs, PRs offer superior resolution, establishing them as a widely utilized technique for the diagnosis and management of PLs [9]. Consequently, identifying PLs on PRs is a conventional procedure for dentists. However, the accuracy of this diagnosis is highly reliant on the dentist’s expertise and experience [10] and is susceptible to fatigue during examination [11].

DL has been proven to provide accurate and rapid detection capabilities in disease diagnosis, which can improve clinical outcomes [12]. Over the past few years, DL techniques, particularly CNNs, have been extensively applied in the research of identifying common oral diseases such as dental caries [13], periodontal disease [14], periapical disease [15], and cracked teeth [16]. Chen [17] et al. employed a deep CNN based on Faster R-CNNs to identify and classify dental caries, periapical periodontitis, and periodontitis across a dataset of 2,900 PRs. The average precision for detecting mild PLs was under 0.2, while moderate lesions exhibited an average precision ranging from 0.2 to 0.3, and severe lesions achieved an average precision between 0.5 and 0.6. However, there are currently relatively few studies validating the impact of CNNs on the diagnosis of PLs by dental professionals. A new study employed a novel 3D deep CNN algorithm named PLA-Net to detect PLs in 4951 CBCT scans of teeth [18]. The algorithm attained an AUC of 0.98, facilitating an enhancement in AUC values for for the diagnosis of PLs across dentists with varying levels of expertise, while concurrently diminishing the time required for diagnosis.

ConvNeXt [19] is an innovative DL architecture for image classification. It was introduced to combine the strengths of both CNNs and transformer-based models, offering a modern and efficient solution to visual recognition tasks. As far as we are aware, no previous studies have reported the use of the ConvNeXt model to assist novice dentists in diagnosing PLs. ResNet34 [20] is a member of the ResNet (Residual Network) family, and its core innovation lies in residual learning. By utilizing skip connections, it enables the training of deeper networks while mitigating the issue of gradient vanishing. This makes it suitable for applications with limited computational resources, such as medical image analysis. Therefore, this study aims to explore the performance comparison between ConvNeXt and ResNet34, in detecting PLs in PRs, and to validate the impact of the ConvNeXt model in assisting novice dental professionals in diagnosing PLs. This research will provide more model options for future studies and accelerate the clinical application of artificial intelligence(AI) in oral medicine.

## Materials and methods

### Model selection

ConvNeXt is a modern CNN proposed by the Facebook AI research team in 2022 [19]. Its design aims to adjust the CNN architecture to achieve performance comparable to or surpassing Vision Transformer while retaining the computational efficiency of CNNs. ConvNeXt is primarily inspired by Swin Transformer, with key innovations including replacing traditional batch normalization with LayerNorm and using larger convolutional kernels (e.g., 7 × 7 instead of ResNet’s 3 × 3), thereby enhancing global feature extraction capabilities. It is well-suited for more complex tasks such as high-resolution medical image analysis and image segmentation.

ResNet, on the other hand, is a deep CNN architecture introduced by Microsoft Research in 2015. Its main innovation is the residual connection, which incorporates skip connections to effectively mitigate gradient vanishing and degradation issues in deep networks, enabling efficient training of deeper models [21]. With lower computational costs, ResNet is suitable for medical image tasks with limited computing resources.

### PRs selection

The PRs used for model training and validation were captured between 2018 and 2023 at Jinan Stomatological Hospital using the periapical bisecting-angle technique. The PRs used in this study followed broad inclusion criteria, excluding only deciduous teeth, after which the images were randomly selected. After manually segmenting individual teeth, a total of 1,305 PRs were collected, including 650 positive for PLs and 655 negative. These images were divided into a training set and a validation set in an 8:2 ratio. A total of 1,044 PRs of individual teeth (520 positive for PLs and 524 negative) were used for model training. To enhance the model’s robustness, data augmentation techniques such as horizontal flipping, exposure adjustment (enhancement/reduction), and sharpening were applied to the 1,044 radiographs, expanding the dataset threefold. Ultimately, 3,132 radiographs were used for model training (1,560 positive for PLs and 1,572 negative). The validation set consisted of 261 PRs and was kept unchanged. During the model-assisted diagnosis phase involving novice dentists, an additional 800 PRs of individual teeth (397 positive for PLs and 403 negative) that were not included in the training or validation sets were selected to assess the model’s impact on the diagnostic performance of novice dentists.

### Auxiliary diagnosis

Three novice dentists were recruited, each with more than one year but less than two years of clinical imaging experience. After training, these novice dentists were able to use the two models to read PRs in a standardized manner. They then independently diagnosed 800 PRs both with and without the assistance of the ConvNeXt and ResNet34 models. In the first round of diagnosis, the three dentists independently identified PLs on the 800 radiographs without any model assistance. To prevent any influence from the first diagnosis, the results were not disclosed to the dentists. In the second round, the ConvNeXt and ResNet34 models were first used to identify PLs on the 800 radiographs, with Grad-CAM visualizing the lesion areas. Then, the three dentists made a second judgment on the 800 images based on the models’diagnostic outputs. The diagnostic tasks were performed independently by the dentists in a standardized radiology reading room. The AUC and diagnostic time of the three dentists, both with and without model assistance, were recorded as evaluation criteria. In this study, all patients involved in the PRs provided informed consent, and the experimental procedures were strictly conducted in accordance with the principles and regulations outlined in the Declaration of Helsinki. Additionally, the three novice dentists provided their signed informed consent.

### PRs segmentation and labeling

The gold standard for PLs diagnosis during model training and validation was established by three oral radiologists with over 15 years of experience. These experts used the image annotation tool LabelImg to label the PLs in individual tooth images, manually annotating regions of interest (ROIs) containing PLs. To ensure the reliability of the standards, the presence of PLs in the images was confirmed only if at least two out of the three experts reached a consensus on the annotation results.

### Evaluation of diagnostic performance

This study employed a comprehensive set of evaluation metrics commonly used in image classification models to compare the effectiveness of ConvNeXt and ResNet34 in identifying PLs. These metrics include precision, F1 score, accuracy, sensitivity, specificity, and AUC. The calculation formulas are presented in Eqs. (1–5).

#### Precision

The proportion of true positives among all samples predicted as positive for PLs.

#### F1 Score

The harmonic mean of precision and recall, serving as an indicator of the equilibrium between a model’s precision and recall.

#### Accuracy

The ratio of correctly classified samples to the total number of samples.

#### Sensitivity

Equivalent to recall, this metric represents the proportion of correctly identified positive cases among all actual positive instances of PLs. Higher sensitivity correlates with a reduced miss rate for positive PLs.

#### Specificity

The proportion of correctly identified negative cases among all true negative instances of PLs. Elevated specificity signifies a lower false positive rate for negative PLs, thus reducing erroneous positive predictions.

#### AUC

This metric is used to quantify the overall effectiveness of a classification model. The AUC ranges from 0 to 1, indicating the model’s capacity to distinguish between positive and negative PLs cases across varying thresholds. A higher AUC value reflects superior model performance, offering a more balanced trade-off between sensitivity and specificity.

1 $$\Pr ecision = \frac{{TP}}{{TP + FP}}$$

2 $$F1 = \frac{{\Pr ecision \times \operatorname{Re} call}}{{\Pr ecision + \operatorname{Re} call}} \times 2$$

3 $$Accuracy = \frac{{TP + TN}}{{TP + TN + FP + FN}}$$

4 $$Sensitivity = \frac{{TP}}{{TP + FN}}$$

5 $$Specificity = \frac{{TN}}{{TN + FP}}$$

In this study, TP (True Positive) denotes the number of samples that are genuinely positive for PLs and are accurately classified as positive by the model. TN (True Negative) refers to the number of samples that are genuinely negative for PLs and are correctly identified as negative by the model. FP (False Positive) indicates the number of samples that are actually negative for PLs but are erroneously predicted as positive by the model. FN (False Negative) refers to the number of samples that are genuinely positive for PLs but are mistakenly predicted as negative by the model.

We compared the diagnostic performance of three novice dentists when using ConvNeXt and ResNet34 models as diagnostic aids. The AUC and diagnostic time (measured in seconds) were recorded under both independent diagnosis and model-assisted diagnosis conditions to evaluate the effectiveness of the two models in assisting diagnosis.

## Results

**Fig. 1:**
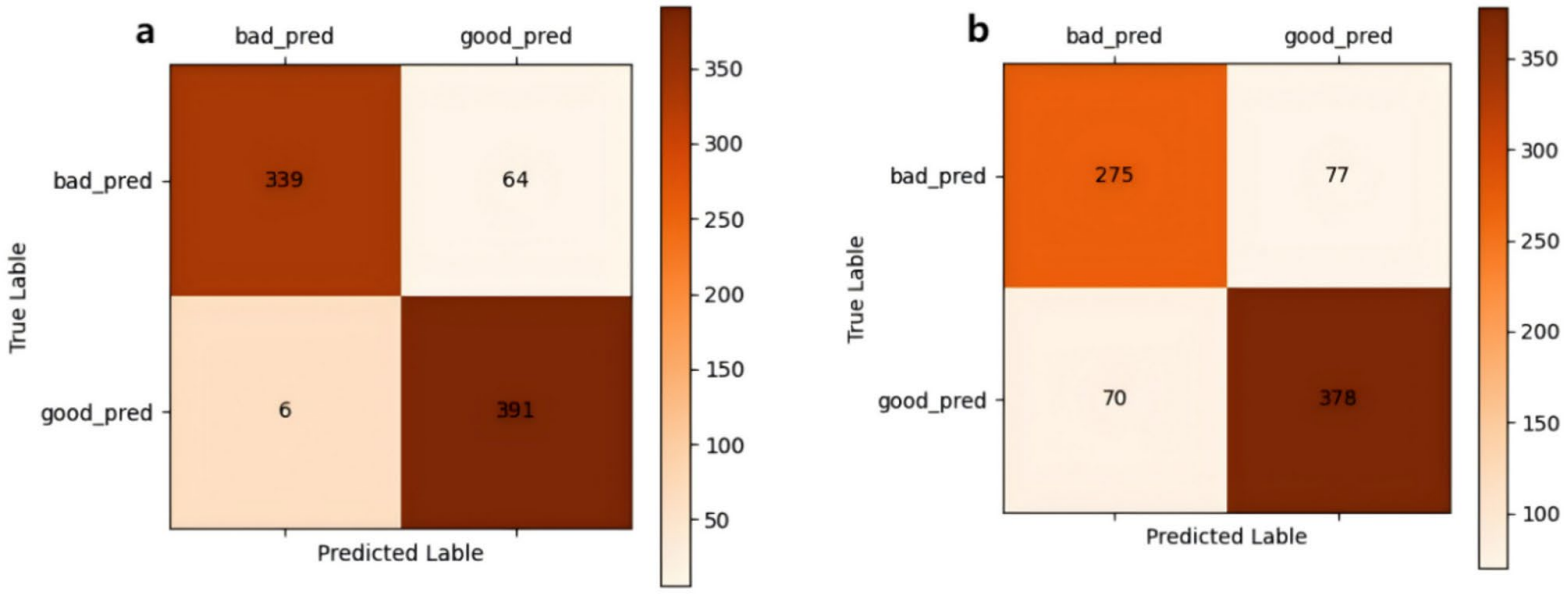
The confusion matrices for ConvNeXt (**a**) and ResNet34 (**b**) are shown in the figure. In (**a**), TN equals 339, indicating that the model correctly predicted 339 PLs negative samples as negative. FP equals 64, meaning the model incorrectly predicted 64 PLs negative samples as positive. FN equals 6, showing that the model incorrectly predicted 6 PLs positive samples as negative. TP equals 391, indicating that the model correctly predicted 391 PLs positive samples as positive. Compared to (**b**) with TN = 275, FP = 77, FN = 70, and TP = 378, (**a**) demonstrates a better accuracy in identifying PLs positives and fewer misclassifications of negatives

During the model comparison phase, both models demonstrated satisfactory performance in detecting PLs. In medical image analysis, confusion matrices can be used to assess the accuracy of models in diagnosing PLs, helping clinicians understand the model’s strengths and limitations and make more informed decisions. Figure 1 shows the confusion matrices for ConvNeXt and ResNet34 models in identifying PLs.

**Fig. 2:**
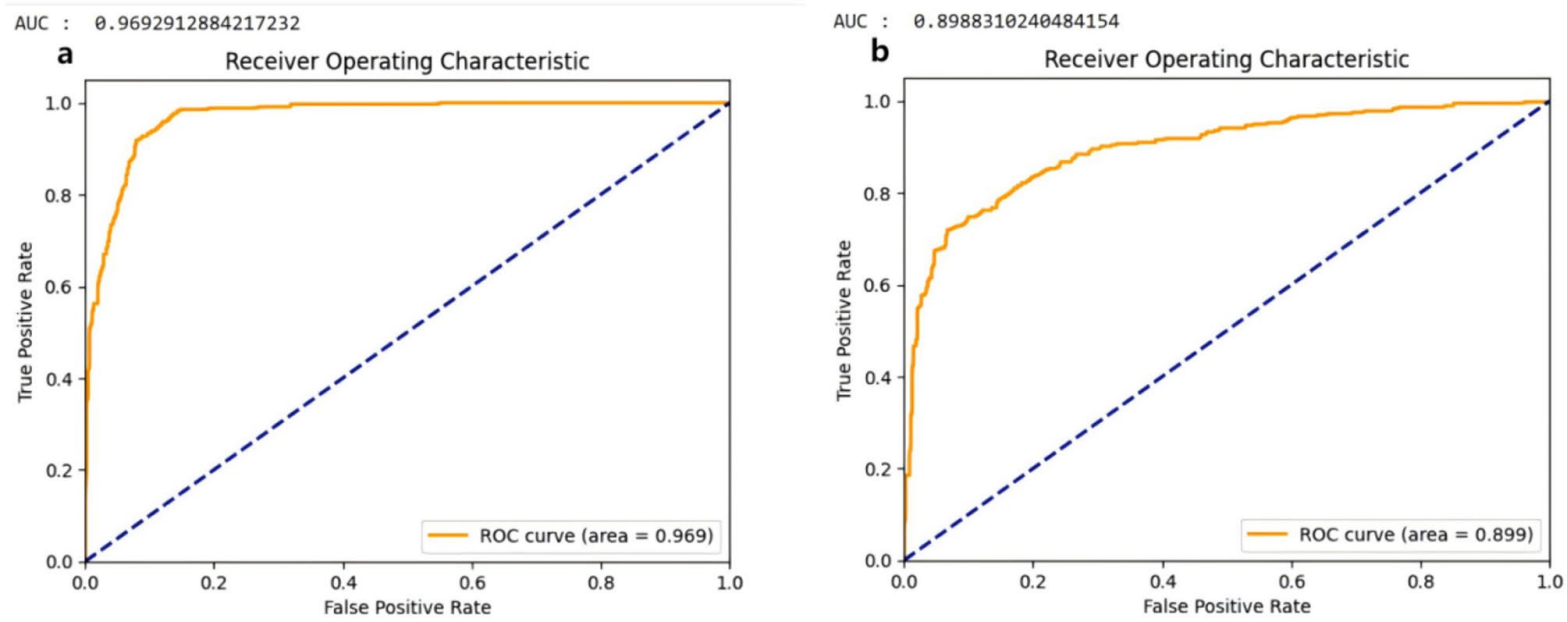
The ROC curves for PL recognition using ConvNeXt (**a**) and ResNet34 (**b**) are shown in the figure. Panel (**a**) demonstrates the characteristics of a high-performance model, with the curve rising sharply and approaching the top-left corner, indicating that ConvNeXt maintains a high true positive rate and a low false positive rate, effectively distinguishing between positive and negative PL samples. In contrast, panel (**b**) shows a slower rise compared to (**a**), with a relatively smoother middle section, suggesting that under certain thresholds, ResNet34 exhibits an increased false positive rate while its improvement in the true positive rate is limited. The performance of the models can be directly evaluated by calculating the AUC. The AUC of the ConvNeXt model (**a**) is 0.9693, while the AUC of the ResNet34 model (**b**) is 0.8988

The ConvNeXt model achieved a precision of 85.93%, an F1 score of 0.92, an accuracy of 91.25%, a sensitivity of 98.49%, a specificity of 84.11%, and an AUC of 0.9693 in identifying PLs from 800 individual teeth PRs. In comparison, the ResNet34 model had a precision of 83.08%, an F1 score of 0.84, an accuracy of 81.63%, a sensitivity of 84.38%, a specificity of 78.13%, and an AUC of 0.8988. The ConvNeXt model outperformed ResNet34 in all metrics, particularly in terms of sensitivity, which is crucial for helping novice dentists detect early and subtle lesions (Table 1, Fig. 2). Performance metrics enable researchers and practitioners to compare and evaluate the performance of models [22] and select the most suitable architecture for the auxiliary diagnosis of PRs.

**Table 1:** ConvNeXt outperforms ResNet34 across all metrics, making it more suitable for identifying PLs. It demonstrates greater reliability and practicality, especially in medical imaging diagnosis where high accuracy and low missed detection rates are essential

	ConvNeXt	ResNet34
Precision	85.93%	83.08%
F1 Score	0.92	0.84
Accuracy	91.25%	81.63%
Sensitivity	98.49%	84.38%
Specificity	84.11%	78.13%
AUC	0.9693	0.8988

During the auxiliary diagnostic phase, the DL model serves as a tool to assist novice dentists in making diagnostic decisions rather than replacing them. To evaluate whether the model enhances diagnostic accuracy, we analyzed the AUC values of three novice dentists across two scenarios: independent diagnosis and model-assisted diagnosis. In the first round, the dentists independently assessed PLs using 800 PRs of individual teeth without model assistance, and their AUC values and diagnostic times were recorded. In the second round, the use of ConvNeXt and ResNet34 models improved the diagnostic performance of all three dentists. With ConvNeXt assistance, the AUC for Dentist A increased from 0.88 to 0.94, for Dentist B from 0.89 to 0.92, and for Dentist C from 0.86 to 0.95, the average AUC of the three dentists increased from 0.88 to 0.94 (Table 2). Additionally, diagnostic time was significantly reduced: Dentist A’s diagnostic time decreased from 169.5 min to 134.8 min, Dentist B’s time decreased from 178.6 min to 135.7 min, and Dentist C’s time decreased from 188.4 min to 155.3 min, with the average diagnostic time reduced from 178.8 min to 141.9 min (Table 3). Similarly, ResNet34 improved Dentist A’s AUC from 0.88 to 0.91, Dentist B’s AUC from 0.89 to 0.92, and Dentist C’s AUC from 0.86 to 0.90, raising the average from 0.88 to 0.91. Diagnostic times were also reduced: Dentist A’s time decreased from 169.5 min to 144.8 min, Dentist B’s time from 178.6 min to 139.6 min, and Dentist C’s time from 188.4 min to 176.3 min, with the average diagnostic time shortened from 178.8 min to 153.6 min. Using the statistical method of independent samples t-test, the results show that ConvNeXt significantaly outperforms ResNet34 in assisting the three doctors to improve AUC (*P* < 0.05).

**Table 2:**
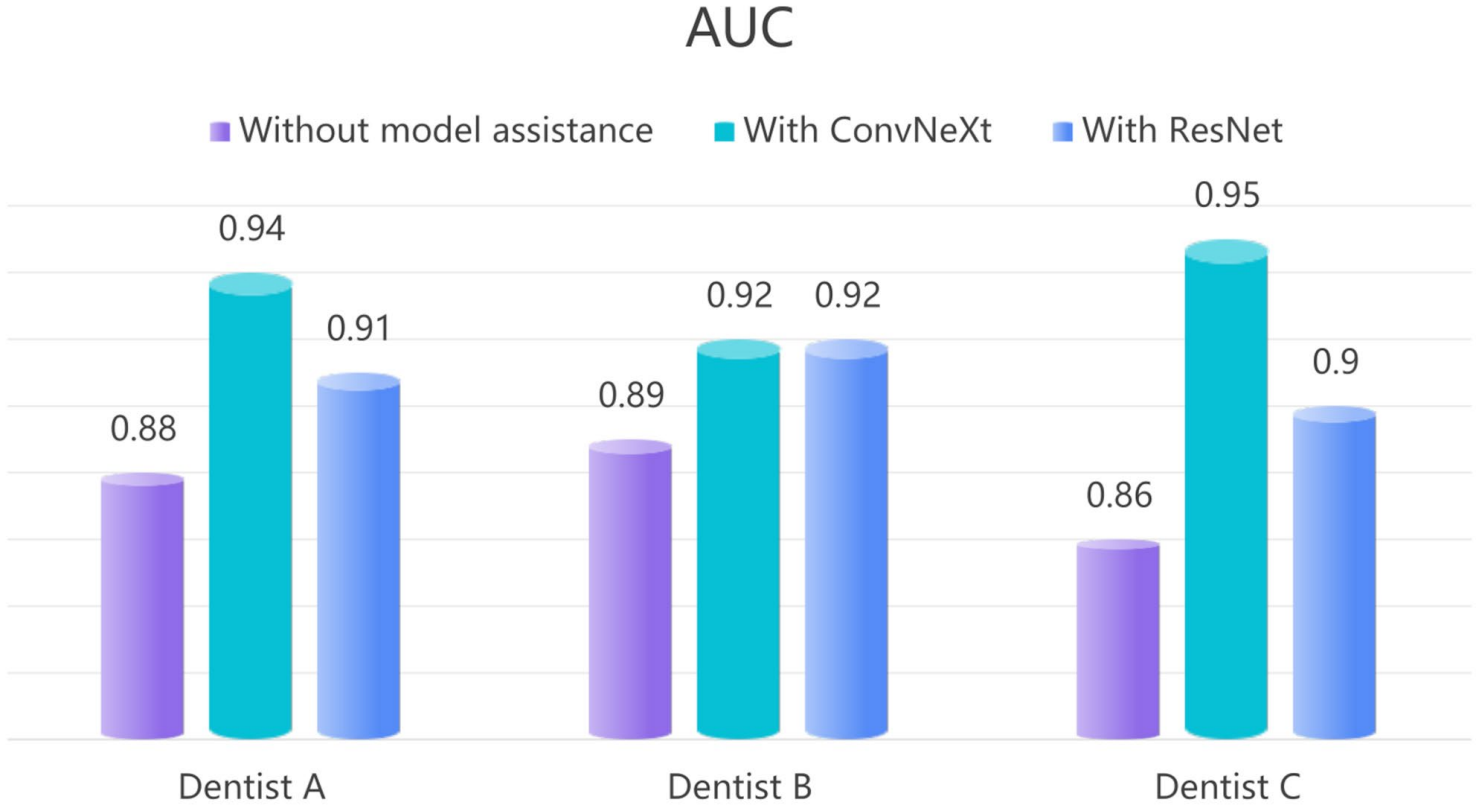
Both models enhanced the diagnostic accuracy of the three novice dentists; however, ConvNeXt significantly outperformed ResNet34 in improving the AUC for all three dentists (*P* < 0.05), particularly for dentist C, whose initial diagnostic accuracy was lower due to limited clinical experience

**Table 3:**
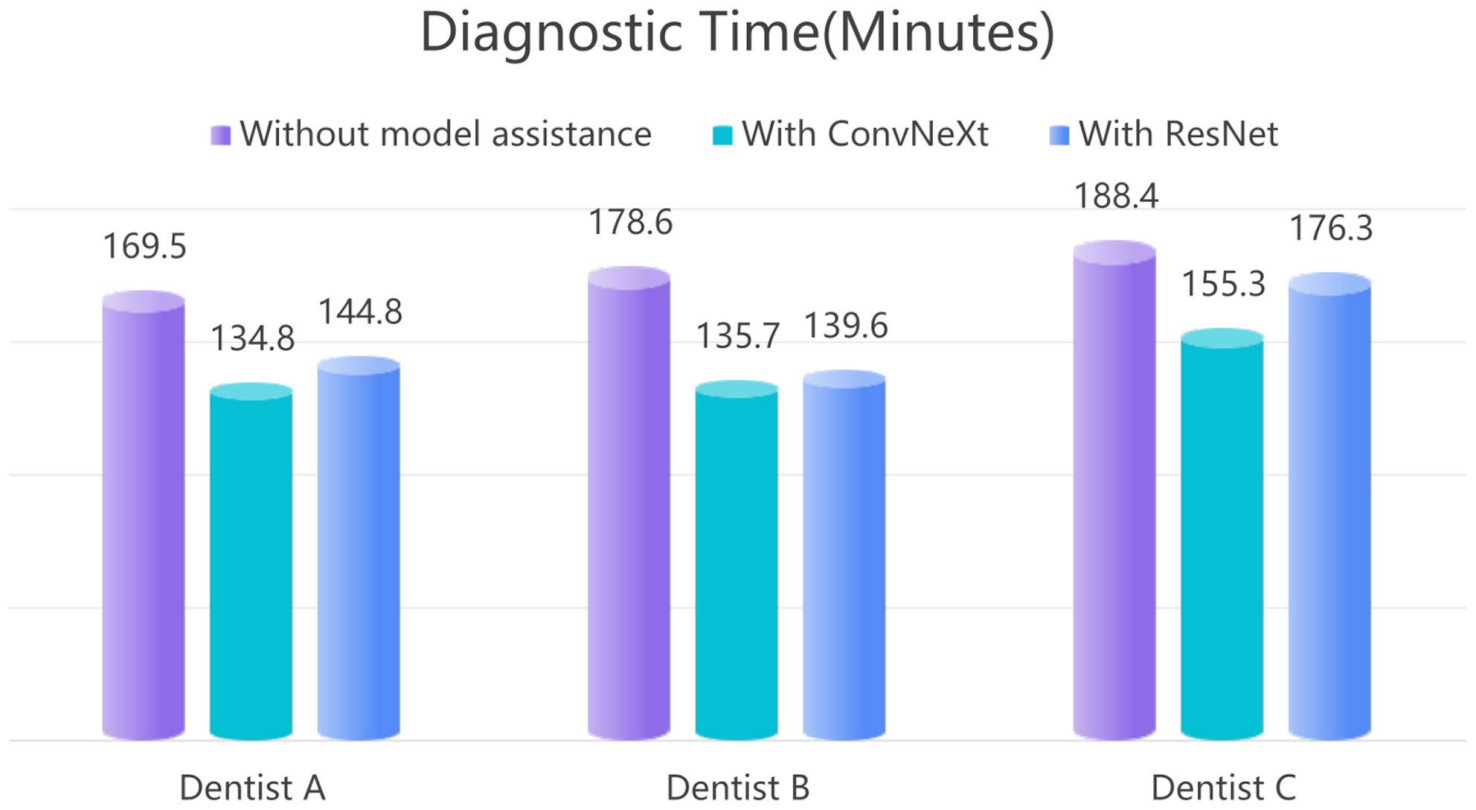
Diagnostic time is also an important metric for evaluating the effectiveness of model assistance. The ConvNeXt model not only enhanced the diagnostic accuracy of the novice dentists but also significantly reduced their diagnostic time, thus improving overall efficiency

**Fig. 3:**
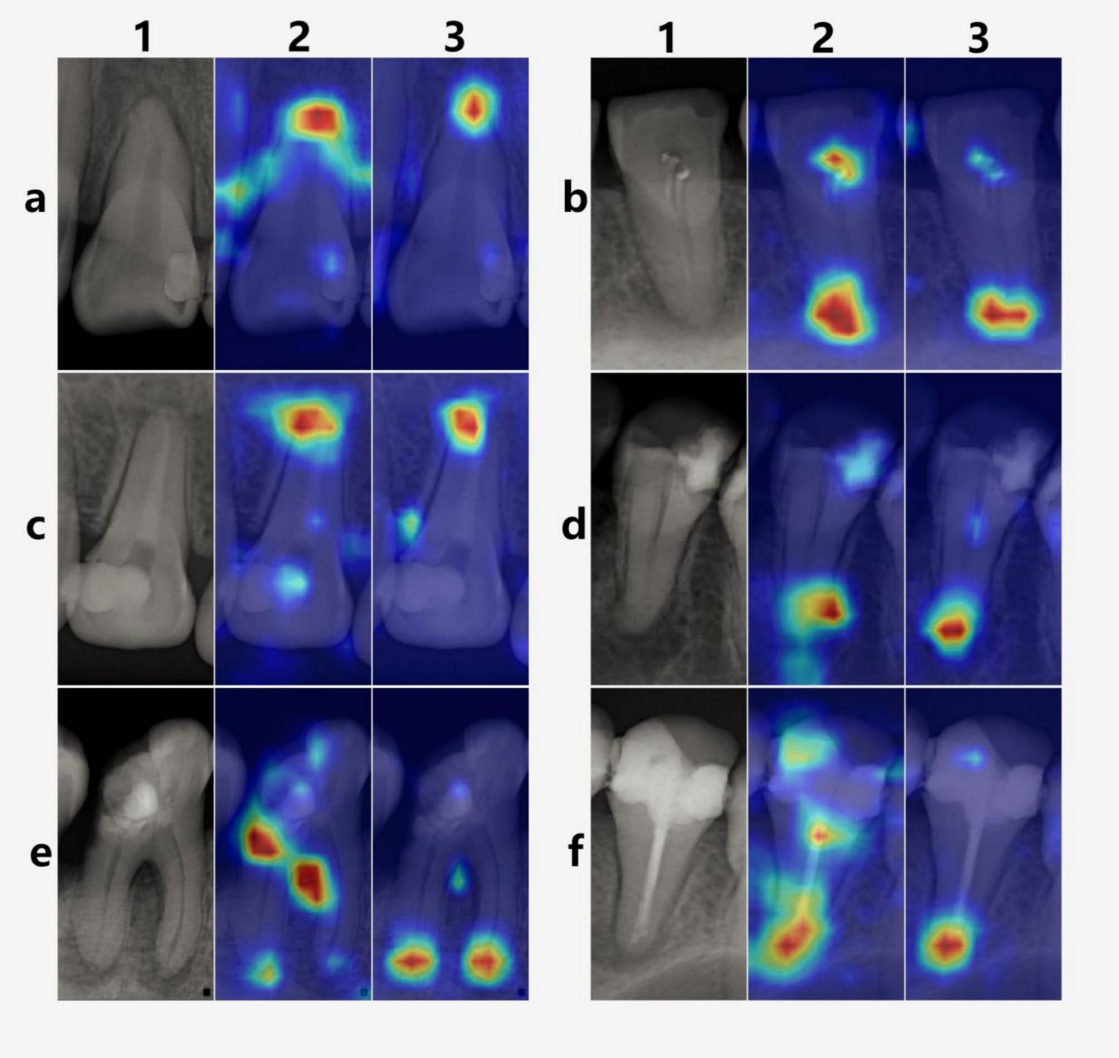
The initial purpose of the study was to evaluate the clinical auxiliary diagnostic capability of the model, so we did not impose overly strict criteria for excluding images. Even when dealing with low-density periodontal structures, crown restorations, and root canal fillings, ConvNeXt was still able to accurately indicate PLs

The partial recognition results of the two models are shown in Fig. 3. Column “1” represents images of individual teeth manually segmented by three experts. Column “2” shows the results identified by the ResNet34 model, while column “3” presents the results identified by the ConvNeXt model. It can be clearly observed that ConvNeXt is less affected by inflammation between tooth roots and periodontal inflammation when identifying PLs, thereby reducing the impact of low-density shadows in other areas (a3, e3). For images containing root canal fillings and crown fillings, the Grad-CAM heatmaps generated by ConvNeXt are more focused and accurate in localization (b3, c3, d3, f3).

## Discussion

The swift advancement of AI has significantly enhanced the analysis of medical imaging across diverse areas, including dentistry, particularly through the application of DL models and CNNs [23]. Research has shown that PLs pose the greatest challenge in studies using DL to identify common dental diseases [24]. In clinical practice, the early identification of PLs, followed by root canal therapy, holds the potential to fully resolve the condition or, at the very least, mitigate the size of the lesion [25]. Therefore, it is of great significance to validate the use of DL models to assist novice dentists in diagnosis. It is worth mentioning that, despite the difficulty in diagnosing PLs in teeth with root canal fillings [26], we did not exclude periapical radiographic images featuring root fillings, root canal treatments, or residual roots and crowns, as such conditions are commonly encountered in clinical practice. The ConvNeXt CNN architecture originates from the latest advancements in computer vision, particularly focusing on the relationship between convolutional networks and self-attention mechanism models. ConvNeXt is based on the classic ResNet architecture and has been improved in line with the Swin Transformer framework [19], incorporating features such as depthwise separable convolutions and replacing traditional BatchNorm with LayerNorm, aimed at enhancing CNN performance. A recent study compared ConvNeXt with other CNN models in classifying dental implant systems using panoramic radiographs [22]. The results showed that ConvNeXt outperformed other CNN models with a high accuracy of 95.74%, demonstrating its potential as an objective and efficient auxiliary diagnostic tool. Another recent study utilized ConvNeXt and other models for the automatic detection of vertical root fractures in intraoral PRs [27]. Due to the fine cracks and low contrast in these images, manually identifying vertical root fractures can be challenging. The results indicated that ConvNeXt and other CNN models could assist dentists in making accurate diagnoses of vertical root fractures. According to our understanding, no studies have yet applied ConvNeXt to assist dentists in detecting PLs. The innovation of this study lies in the first-ever use of the advanced ConvNeXt model to assist novice dentists in diagnosing PLs. This research validates the role of DL models in supporting human doctors’ diagnostic efforts and provides a model selection reference for future clinical applications of artificial intelligence. Furthermore, in this study, we compared the ConvNeXt model with the ResNet34 model, which has been well-established for classification tasks on PRs [28], demonstrating ConvNeXt’s immense potential for future DL research in disease diagnosis. Most importantly, this study validates that the ConvNeXt model can improve the diagnostic performance of novice dentists in detecting apical lesions on PRs, while significantly reducing diagnosis time, which could bring considerable social and economic value to clinical practice. One of the limitations of this study is that the segmentation of individual teeth in the PRs was done entirely manually. This significantly increased the time cost, especially for large-scale datasets, where annotation efficiency is very low. However, models typically require large-scale, high-quality annotated data to achieve good generalization capabilities, and insufficient data may result in suboptimal performance in test sets or practical applications. Additionally, issues of subjectivity and consistency arise, making automation difficult to achieve. We drew inspiration from two studies that successfully applied U-Net for the segmentation of PLs [29, 30]. U-Net is a DL framework primarily used for image segmentation. In future research, using an image segmentation architecture or object detection algorithms combined with image classification models could address the limitations of manual segmentation. Another limitation of this study is that it only collected PRs from Jinan Stomatological Hospital, without conducting a multi-center study. In current research, medical institutions serve as the primary sources of data for model training; however, privacy issues prevent data sharing between different centers. Consequently, datasets are often sourced from a single institution, resulting in smaller sample sizes and potential biases in data distribution. This significantly increases the risk of overfitting in models developed using such data and may reduce the objectivity of the model evaluation results. In future research, we plan to broaden data collection by acquiring PRs from multiple medical institutions using various X-ray devices. This will be followed by standardized preprocessing to enhance the model’s robustness. Furthermore, given the overlap of adjacent anatomical structures and the presence of numerous low-density confounding features in the apical region, such as the maxillary sinus and mental foramen, a single detection model directly analyzing PRs may struggle to accurately identify PLs. Segmenting individual tooth images can help minimize interference from surrounding tissues, and DL models are capable of performing this segmentation automatically, thereby increasing efficiency. Consequently, a primary objective of future research will be to develop a diagnostic approach that combines automatic segmentation of individual teeth in PRs with precise detection of PLs. The potential of AI and DL in dentistry presents a promising area for exploration, with ongoing advancements aimed at enhancing diagnostic accuracy and decision-making support for clinicians.

## Conclusion

This study validated the excellent performance of the DL model ConvNeXt in assisting novice dentists with diagnosing PLs in PRs. It filled a gap in the application of ConvNeXt for the auxiliary diagnosis of PLs and provides a reference for model selection in future AI applications in clinical dentistry.

## Data Availability

The datasets generated during and/or analyzed during the current study are available from the corresponding author on reasonable request.

## Abbreviations

CNN: Convolutional Neural Network
PLs: Periapical Lesions
DL: Deep Learning
PRs: Periapical Radiographs
AUC: Area Under the Curve
CBCT: Cone Beam Computed Tomography
